# Supplementary material for: Three-dimensional character of the deformation twin in magnesium
Source: Nat Commun. 2019 Jul 25;10:3308. doi: 10.1038/s41467-019-10573-7 (PMC6658514; doi:10.1038/s41467-019-10573-7)
Supplement: Supplementary file 1 — Supplementary Information [file 41467_2019_10573_MOESM1_ESM.pdf]

Supplementary Information

Three-dimensional character of the deformation twin in magnesium

Liu et al.

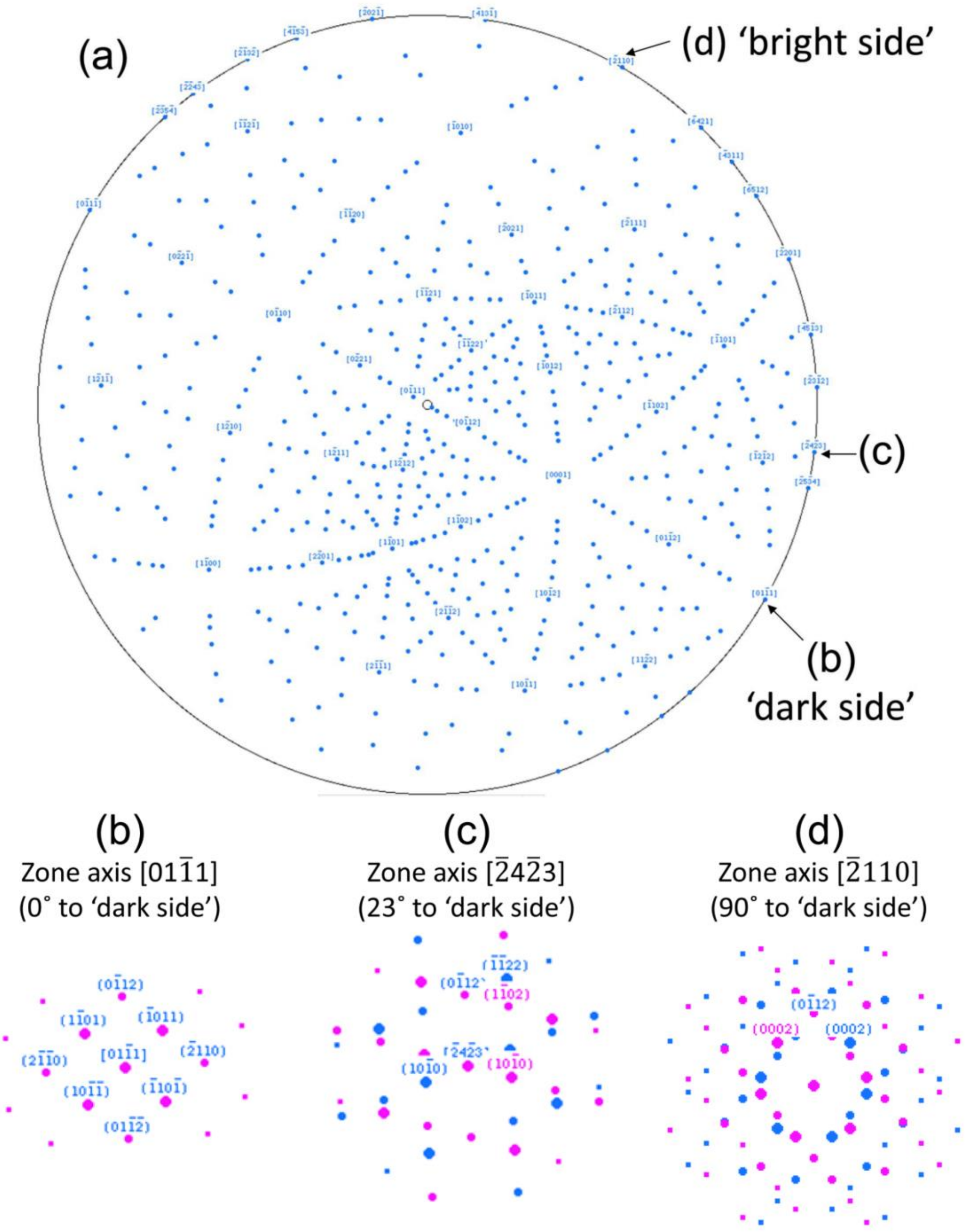

Supplementary Figure 1. Twinning plane stereogram observing in the twinning plane normal (TPN) view. (a) $(0\bar{1}12)$ twinning plane stereogram for magnesium (Mg), where several low-index zone axes near the periphery are identified: (b) $[01\bar{1}1]$ ('dark side'); (c) $[\bar{2}4\bar{2}3]$; and (d) $[\bar{2}110]$. Twin and matrix (pink and blue symbols, respectively) diffraction patterns overlap when observing along $[01\bar{1}1]$, but can be identified along both $[\bar{2}4\bar{2}3]$ and $[\bar{2}110]$ zone axis.

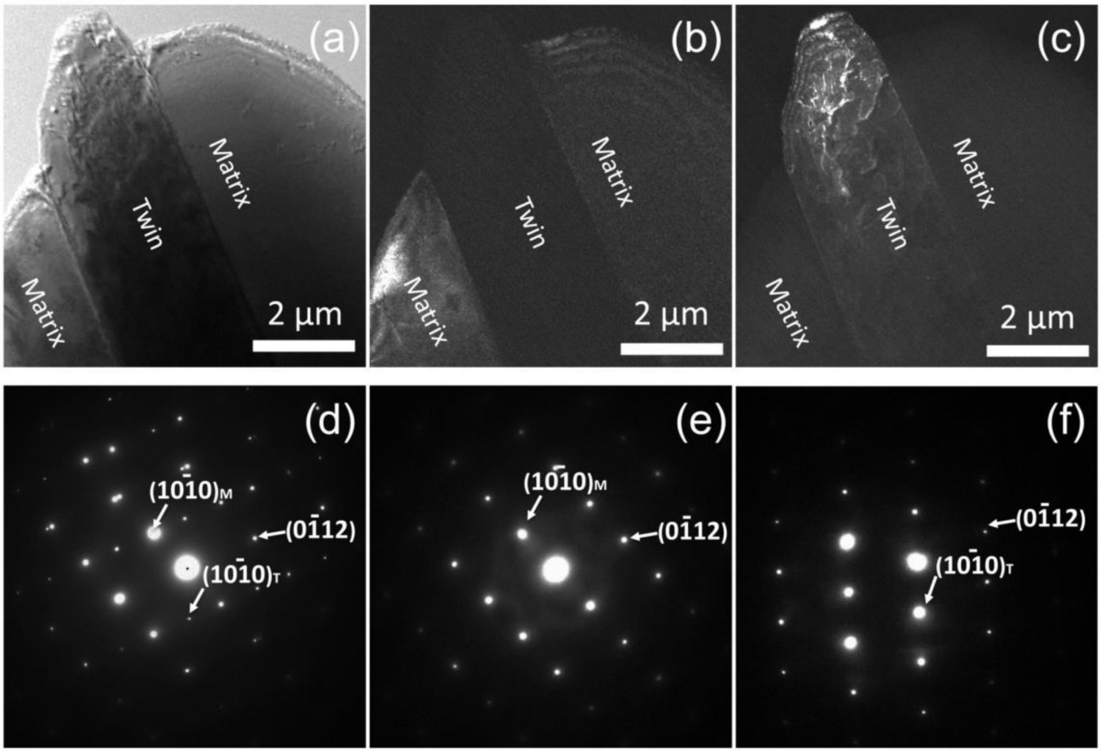

Supplementary Figure 2. Transmission electron microscopy (TEM) images and corresponding selected-area diffraction (SAD) patterns observing along [$\bar{2}4\bar{2}3$] zone axis. (a) Bright-field TEM, dark-field TEM of (b) matrix domains and (c) a twin domain, and (d) SAD patterns for entire area reveal the twin relation when observing along [$\bar{2}4\bar{2}3$]. Moreover, SAD patterns of (e) a matrix domain and (f) a twin domain can be identified, respectively.

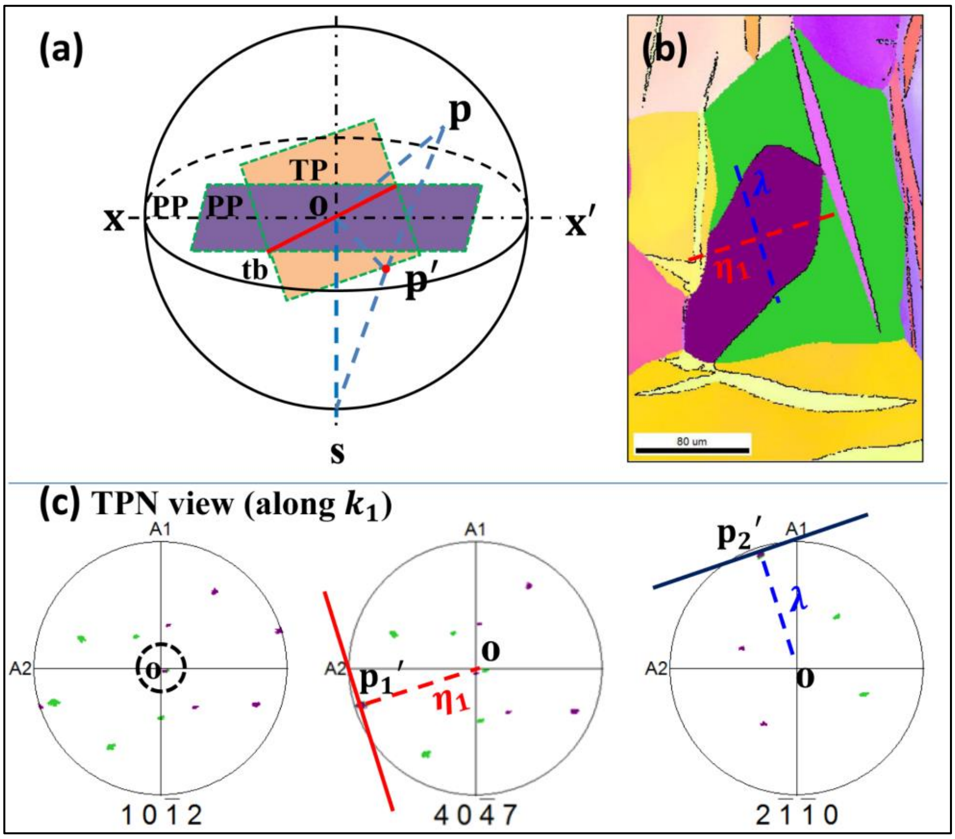

Supplementary Figure 3. Method to determine twin relation and crystallographic directions by electron back-scatter diffraction (EBSD) pole figures. Crystallographic plane trace and plane normal can be projected on pole figures as line and point, respectively. (a) **TP** is the inclined twinning plane and **PP** is the projection plane, **tb** is the intersecting line between **TP** and **PP**. **op** is the **TP** plane normal and **p** intersects the sphere. If one connects points **s** and **p** (both on the sphere), $\boldsymbol{p'}$ is the intersecting point of **op** on **PP** plane. According to geometry, since **os**⊥**PP**, thus **os**⊥**tb**. (color in red). Also, since **op**⊥**TP**, thus **op**⊥**tb.** Because **tb**⊥**op** and **tb**⊥**os**, **tb**⊥**ops** (the plane including both **op** and **os**), therefore **tb**⊥$\boldsymbol{op'}$ (one line on **ops** plane). (b-c) An irregular shaped deformation twin (color in purple) and its pole figures of twin and matrix. This twin is in the TPN view from $\{10\bar{1}2\}$ pole figure (black dashed circle). Based on the analysis in (a), $\boldsymbol{op_1}'$ is parallel to <$10\bar{1}1$> ($\boldsymbol{\eta_1}$, and also the $\{40\bar{4}7\}$ plane normal), and $\boldsymbol{op_2}'$ is parallel to <$2\bar{1}\bar{1}0$> ($\boldsymbol{\lambda}$, and also the $\{2\bar{1}\bar{1}0\}$ plane normal). Therefore, crystallographic directions ($\boldsymbol{\lambda}$ and $\boldsymbol{\eta_1}$) can be marked in (b).

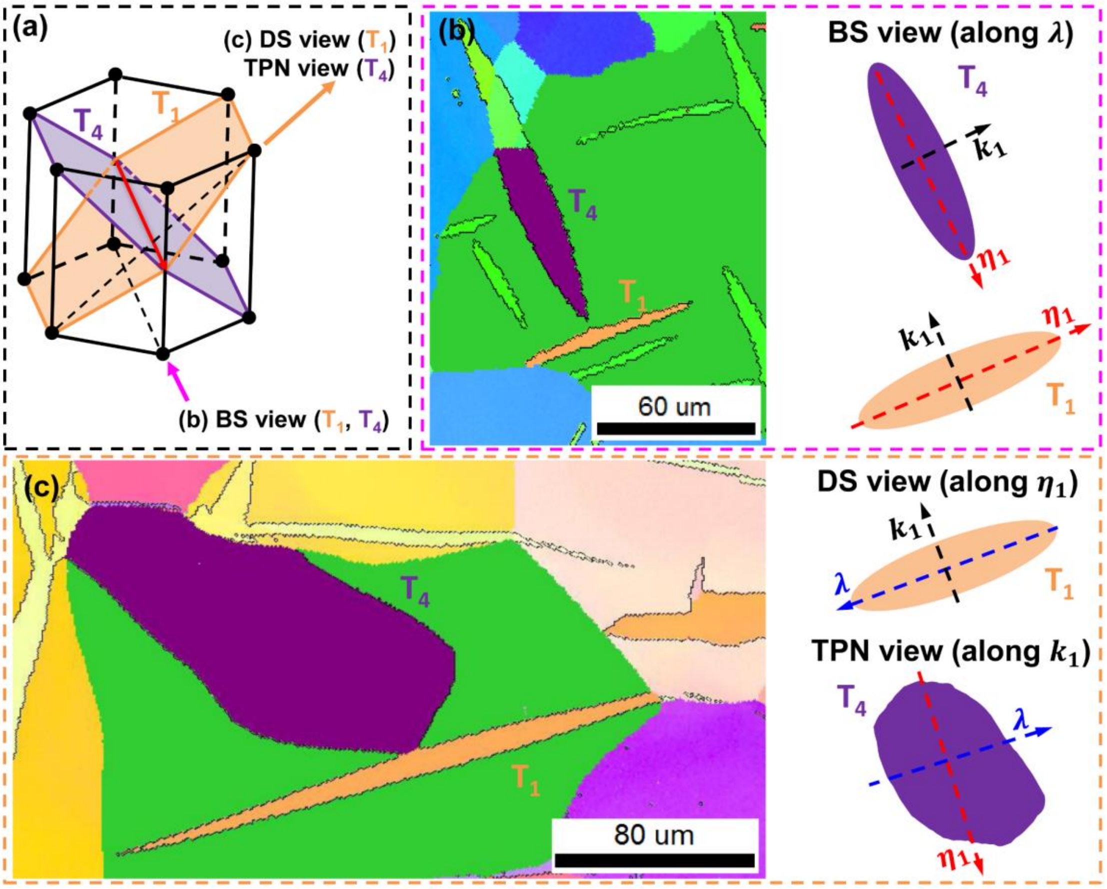

Supplementary Figure 4. Showcase of the irregular twin shape from TPN view compared to lenticular twin shapes from bright side (BS) and dark side (DS) view. (a) Typical co-zone twinning pair (**T**$_1$ and **T**$_4$) with the red intersection line parallel to $[2\bar{1}\bar{1}0]$. Twin sections from (b) BS view (**T**$_1$ and **T**$_4$) and (c) DS view (**T**$_1$) showing lenticular shapes with short axis along $\boldsymbol{k_1}$ and long axis along $\boldsymbol{\eta_1}$ and $\boldsymbol{\lambda}$, respectively. By comparison, (c) TPN view (**T**$_4$) showing an irregular shape.

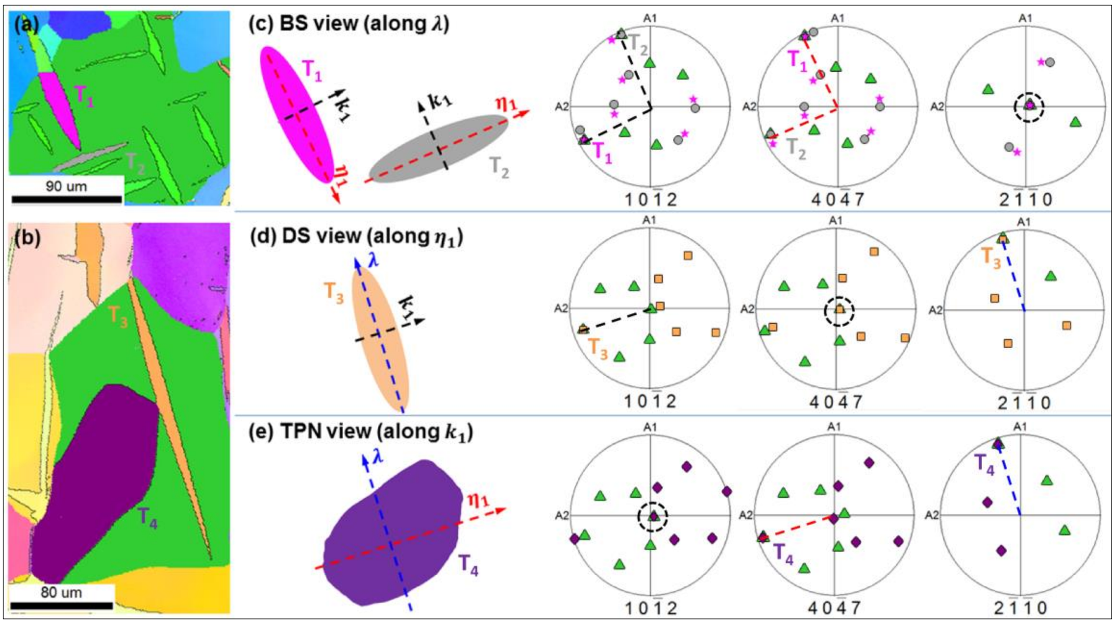

Supplementary Figure 5. Detailed analysis of various twin shapes and their corresponding crystallographic directions from BS view, DS view and TPN view. (a) Deformation twins according to BS view (**T_1** and **T_2**), (b) DS view (**T_3**) and TPN view (**T_4**). Their corresponding $\{10\bar{1}2\}$, $\{40\bar{4}7\}$ (plane normal: <$10\bar{1}1$>), $\{2\bar{1}\bar{1}0\}$ pole figures are used to determine intersecting traces of $\boldsymbol{\lambda}$, $\boldsymbol{\eta_1}$, and $\boldsymbol{k_1}$. Their schematic showing: (c) for BS view, $\boldsymbol{\eta_1}$ is along long axis and $\boldsymbol{k_1}$ is along short axis, (d) for DS view, $\boldsymbol{\lambda}$ is along long axis and $\boldsymbol{k_1}$ is along short axis, and (e) for TPN view, there is no apparent relation between the shape and the crystallographic directions $\boldsymbol{\lambda}$ and $\boldsymbol{\eta_1}$.

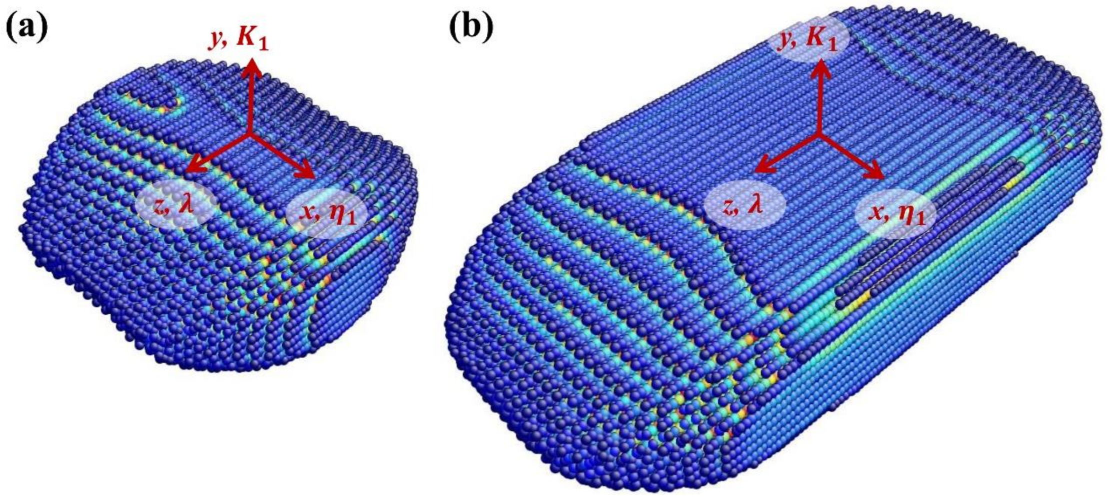

Supplementary Figure 6. Three-dimensional (3D) atomic configuration of twin nucleus before or after loading. (a) 3D atomic configuration of the twin nucleus. (b) Under loading, the twin nucleus grows faster along $\boldsymbol{\lambda}$ direction than along $\boldsymbol{\eta_1}$ direction.

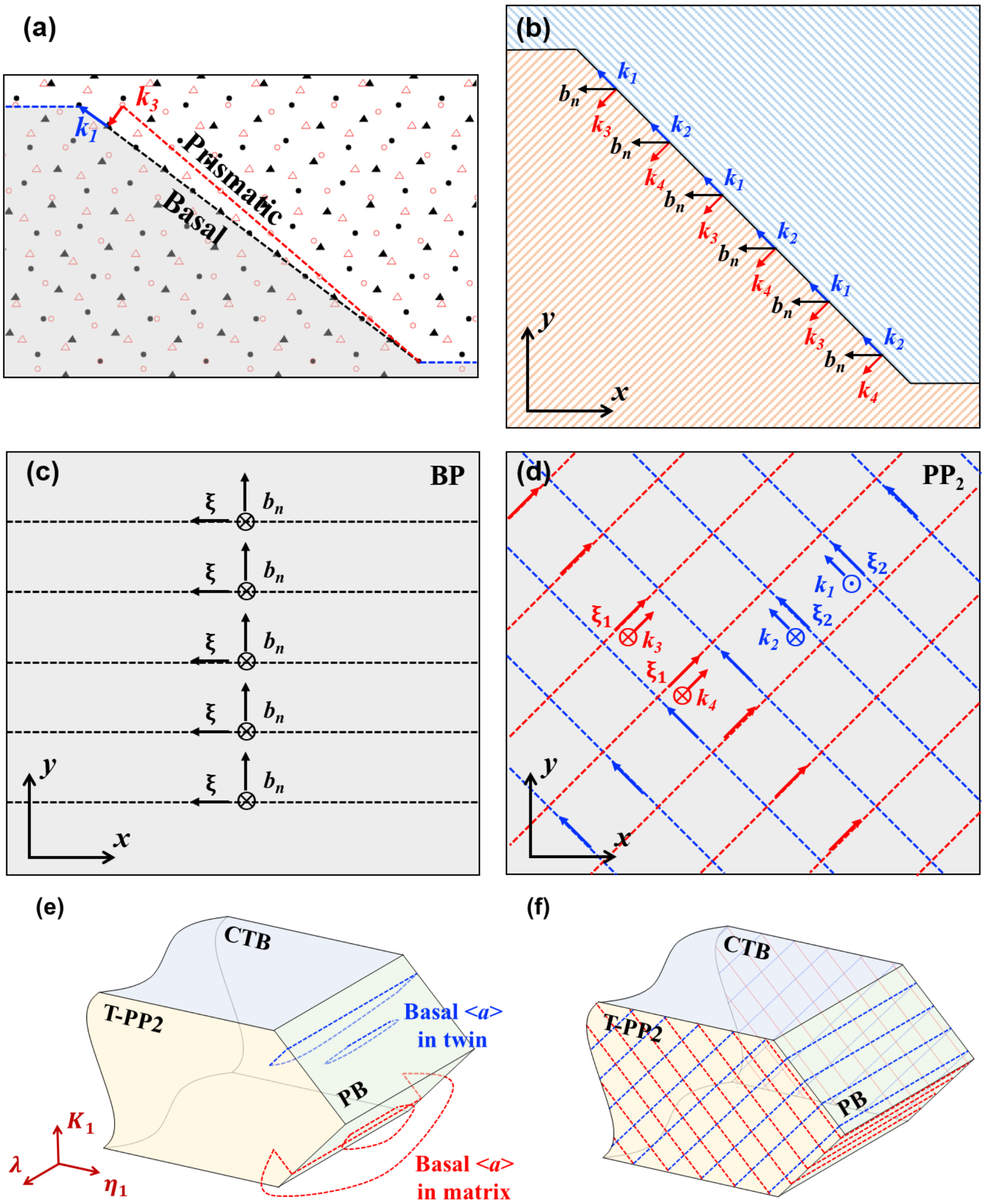

Supplementary Figure 7. Relaxation of twinning boundaries (TBs) to reduce elastic distortion associated with the pileup of twinning dislocations (TDs) and pinning effect of misfit dislocation. (a) Mismatches between basal plane in matrix/twin and prismatic plane in twin/matrix is described by a wedge component $\boldsymbol{k}_3$ and a glide component $\boldsymbol{k}_1$. (b) Non-equilibrium TBs in Mg are relaxed by the emission of <a> lattice dislocations, accompanied by the creation of misfit dislocations with opposite signs on TBs. (c-d) the misfit dislocations pileup condition in the (c) BS view and (d) DS view. (e-f) 3D schematic suggesting two set of misfit dislocations in the DS view may provide more pinning effect.

Supplementary Note 1

Identifying possible low-index crystallographic facets. From twinning plane pole figure and stereogram, **supplementary Fig. 1** showing a $(0\bar{1}12)$ twinning plane stereogram for magnesium, where several low-index zone axes containing the $(0\bar{1}12)$ diffraction vector are identified around the periphery. The simulated diffraction patterns, where the twin and matrix diffraction spots are depicted pink and blue, respectively, overlap exactly when observing from the dark side view. By comparison, separate twin and matrix spots can be identified, for example, along $[\bar{2}4\bar{2}3]$ (Also confirmed experimentally in **Supplementary Fig. 2**) and $[\bar{2}110]$ zone axis. Furthermore, several other low-index directions are identified: $[\bar{2}3\bar{1}2]$, $[\bar{2}201]$, $[\bar{4}311]$. Their corresponding perpendicular planes are then calculated based on the criteria [1], and marked in **Fig. 1a** as $(\bar{8}\ 16\ \bar{8}\ 21)$, $(\bar{4}\ 6\ \bar{2}\ 7)$, $(\overline{17}\ 17\ 0\ 5)$, $(\overline{16}\ 12\ 4\ 7)$. Since there is no reported experimental evidence to confirm this facet, we then pick $(\bar{4}6\bar{2}7)$ as an example and mark it in **Fig. 1e**.

Supplementary Note 2

Formation and influence mechanisms of misfit dislocations. The faster propagation along $\boldsymbol{\eta_1}$ direction than that along $\boldsymbol{\lambda}$ direction observed in **Fig. 4d** when $\boldsymbol{\lambda}$ is larger than 40 μm may be related to the propagation of semi-coherent facets. Structural information such as network of misfit dislocations are likely to affect motion of facets. Twin nuclei, whether formed homogeneously or heterogeneously, are bounded by semi-coherent Prismatic||Basal (BP/PB) interfaces [2,3] with large coherency stresses. The coherent dichromatic complex (CDC) for a $(\bar{1}012)$ twin viewed along $[1\bar{2}10]$ in **Supplementary Fig. 7a,** shows an inclined interface that bonds basal and prismatic planes, referred to as BP or PB facets. The order has the meaning that the first character, B or P, represents the twin plane at the interface and the second character represents the matrix plane. The crystallographic mismatch is described as a Frank vector, associated with the BP or PB step, which has disclination dipole character with an associated rotation angle of $\varphi = 3.71°$ for Mg [4,5]. In order to reduce the local stress fields associated

with the Frank vector **b**, the rotation must be partitioned. Partitioning into two mixed disclinations with both wedge component $\boldsymbol{k_3}/\boldsymbol{k_4}$ and a glide component $\boldsymbol{k_1}/\boldsymbol{k_2}$ reduces the local stress fields. For a twin domain embedded in a matrix, a twin boundary with a regular array of TDs is at non-equilibrium[6]. When the twin grows large enough, non-equilibrium TBs may relax to reduce elastic distortion associated with the pileup of TDs. With minimum twin height, the stresses can be relaxed by the addition of misfit partial dislocations. Relaxation of TBs is accompanied by formation of misfit dislocations that are a result of emission of <a> lattice dislocations (due to its low activation energy and high mobility) into matrix and/or twin domains. As a result, dislocation content in the TB changes and the net Burgers vector decreases, comprising TDs and misfit dislocations. For example, in the coordinate system shown in **Supplementary Fig. 7b** (x-axis along $[10\bar{1}1]$, y-axis normal to $(\bar{1}012)$ plane and z-axis along $[1\bar{2}10]$), a coherent BP converts into a semi-coherent BP by emission of -$\boldsymbol{k_1}$ and -$\boldsymbol{k_2}$ into matrix and -$\boldsymbol{k_3}$ and -$\boldsymbol{k_4}$ into twin, leaving misfit dislocations $\boldsymbol{k_1} = \left(-\frac{\sqrt{6}a}{4}, \frac{\sqrt{6}a}{4}, \frac{a}{2}\right)$, $\boldsymbol{k_2} = \left(-\frac{\sqrt{6}a}{4}, \frac{\sqrt{6}a}{4}, -\frac{a}{2}\right)$, $\boldsymbol{k_3} = \left(-\frac{\sqrt{6}a}{4}, -\frac{\sqrt{6}a}{4}, -\frac{a}{2}\right)$ and $\boldsymbol{k_4} = \left(-\frac{\sqrt{6}a}{4}, -\frac{\sqrt{6}a}{4}, \frac{a}{2}\right)$ on BP facet. The line sense of $\boldsymbol{k_1}$, $\boldsymbol{k_2}$, $\boldsymbol{k_3}$ and $\boldsymbol{k_4}$ is the same. So, $\boldsymbol{k_1}$, $\boldsymbol{k_3}$ and $\boldsymbol{k_2}$, $\boldsymbol{k_4}$ react to form $\boldsymbol{b_n} = \left(-\frac{\sqrt{6}a}{2}, 0, 0\right)$, which enables synchroshear of TDs and misfit dislocations on semi-coherent BP interface [6]. In **Supplementary Fig. 7c**, alignment of misfit dislocation $\boldsymbol{b_n}$ on BP facet is displayed in the coordinate system with x-axis along $[\bar{1}2\bar{1}0]$ direction, y-axis along $[0001]$ direction in twin, and z-axis normal to BP interface. Therefore, these misfit dislocations nucleate at BP steps and loop the 3D twin. Furthermore, there also exists PB facet that is orthogonal to BP facet. Their lines on each BP or PB facets are parallel to TDs, but intersect with TDs on lateral TB. From the DS view, a semi-coherent twist $\{2\bar{1}\bar{1}0\}$ Prismatic||Prismatic (T-PP) interface is shown to have lower interface energy and should be dominant when twin is large [7]. In **Supplementary Fig. 7d**, the same $\boldsymbol{k_1}$, $\boldsymbol{k_2}$, $\boldsymbol{k_3}$ and $\boldsymbol{k_4}$ misfit dislocations pileup on T-PP interfaces. However, because the line directions of $\boldsymbol{k_1}$, $\boldsymbol{k_3}$ and $\boldsymbol{k_2}$, $\boldsymbol{k_4}$ are not parallel to each other, synchroshear cannot take place, which may largely slow down the mobility

of semi-coherent T-PP interface. When twin is large and is not confined by grain boundaries, mobility of facets possibly depends on whether synchroshear occurs for that facet. 3D schematic in **Supplementary Fig. 7e-7f** suggesting two set of misfit dislocations on the DS may provide more pinning effect than on the BS. This may explain the faster propagation along $\boldsymbol{\eta_1}$ direction than that along $\boldsymbol{\lambda}$ direction when $\boldsymbol{\lambda}$ is larger than 40 μm.

Supplementary References

1. C. Frank F. On Miller–Bravais indices and four-dimensional vectors. *Acta Cryst* 1965, **18**(18)**:** 862-866.
2. Wang J, Yadav SK, Hirth JP, Tomé CN, Beyerlein IJ. Pure-Shuffle Nucleation of Deformation Twins in Hexagonal-Close-Packed Metals. *Materials Research Letters* 2013, **1**(3)**:** 126-132.
3. Liu BY, Wang J, Li B, Lu L, Zhang XY, Shan ZW*, et al.* Twinning-like lattice reorientation without a crystallographic twinning plane. *Nature Communications* 2014, **5**(2)**:** 3297.
4. Hirth JP, Wang J, Tomé CN. Disconnections and other defects associated with twin interfaces. *Progress in Materials Science* 2016, **83:** 417-471.
5. Barrett CD, Kadiri HE. The roles of grain boundary dislocations and disclinations in the nucleation of {1 0 1¯ 2} twinning. *Acta Materialia* 2014, **63:** 1-15.
6. Gong M, Hirth JP, Liu Y, Shen Y, Wang J. Interface structures and twinning mechanisms of twins in hexagonal metals. *Materials Research Letters* 2017, **5:** 1-16.
7. Liu Y, Li N, Shao S, Gong M, Wang J, Mccabe RJ*, et al.* Characterizing the boundary lateral to the shear direction of deformation twins in magnesium. *Nature Communications* 2016, **7:** 11577.
